# Cross-discipline collaboration in anthropology and dentistry: an interview with Cinzia Fornai on how collaborations can forge new careers

**DOI:** 10.1038/s42003-021-02771-1

**Published:** 2021-10-28

## Abstract

Cinzia Fornai is a Scientific Coordinator with the Vienna School of Interdisciplinary Dentistry, and was previously a postdoctoral fellow at the Institute of Evolutionary Medicine, University of Zurich. Her current role is focussed more on clinical research than her previous academic positions, and was facilitated by strong interdisciplinary collaboration.

**Figure:**
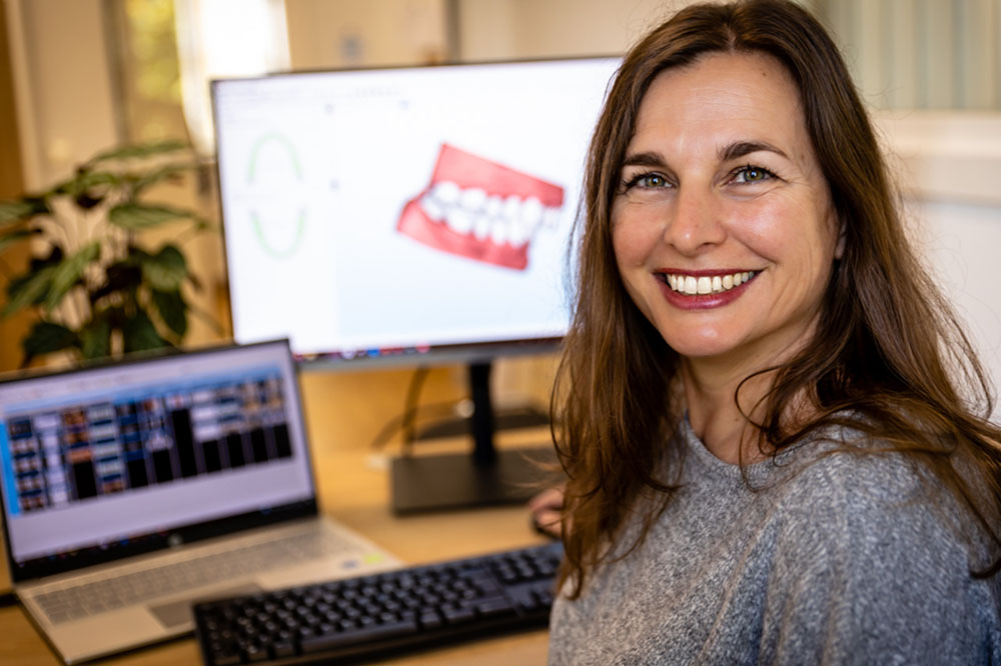
Cinzia Fornai

Please tell us about your research interests!

Building upon my background as evolutionary anthropologist and morphometrician, I have naturally developed an interest in the function of the masticatory system, and in the influence of diet and, more generally, culture and technology on the function and health of the oral structures. I am inclined to interpret modern orofacial ailments as diseases of civilization, thus I am interested in developing projects within the field of evolutionary medicine, with the main goal to elucidate clinically relevant matters. Some particular topics I want to investigate, currently highly debated in Dentistry, are the role of dental occlusion in the etiopathology of orofacial dysfunctions and the effect of malocclusion on the individual chewing efficiency and general well-being.

Which, according to you, is the most exciting recent discovery in evolutionary anthropology?

Most of all, I enjoy observing how the recent paleoanthropological evidence makes us aware of the complexity of the human evolutionary scenario, and pushes us to abandon dogmatic views and rigid classification schemes. I was lucky to join Professor Israel Hershkovitz´s team, studying the Levantine Nesher Ramla findings, and I found it very entertaining to observe how these fossils perfectly fitted the complex puzzle of the Middle Pleistocene *Homo* evolution.

As an anthropologist by training, how has collaborating within Dentistry shaped your career?

My involvement within the field of Dental Medicine has been crucial for me in many regards. First of all, I started to truly appreciate how the understanding of the past influences our interpretation of the present. On a personal level, I was pleased to see how my knowledge and expertise could benefit other disciplines. I also realized that doing clinical research is extremely rewarding, and feeling useful to other human beings is highly motivational. It is imperative for me to add that thanks to the collaboration with dentists I could financially sustain myself and my son during my PhD studies. Thus, I deeply value the open-mindedness and generosity of Professor Rudolf Slavicek (renowned Austrian dentist) who initiated this collaboration.

Many fields are currently developing similar collaborations with dentists, particularly engineers. Why are these collaborations emerging now, rather than before?

The human masticatory system is complex and so is the relationship between its function and morphology. The pathologies affecting it are multifactorial, making it difficult to disentangle the role of the different variables. Consequently, multidisciplinary research is needed to address such challenging topics. We must also consider that the field of Dentistry makes use of high-end materials, instruments and technologies for the diagnosis and the treatment of patients. Thus, it is to be expected that it advances together with other disciplines, such as technology, biomechanics, engineering, morphometrics and, of course, anthropology.

What are the key differences in your new non-academic role, compared to your experiences in academia?

As Scientific Coordinator of the Vienna School of Interdisciplinary Dentistry, my main role is to manage projects while carrying out my own research, therefore my tight relationship with academia is instrumental. I am still affiliated with and teaching at the University of Vienna and University of Zurich, and count on many of my former collaborators for the new projects. Fortunately, I could transfer my knowledge, skills and research ideas directly into the new working environment. Besides that, having a permanent contract, I can now focus on my work rather than on the continuous and strenuous search for a job. Also, I now manage to measure my productivity on a daily basis which results in a sense of accomplishment that was hard to feel when my finish line was the end of a research project.

Is there any advice you would give to researchers looking to make a similar transition?

We often struggle to see how, from our very specialized field of study, we could ever find a job out of academia. I knew there were opportunities for me in the “real World” (to quote my colleague Dr Elisabeth Oberzaucher), but how could I employ my competence and expertise? I am aware now that researchers can make a great contribution in many different environments. Academia is extremely competitive; thus, researchers are well trained and acquire skills highly valued elsewhere. We are hard workers, resilient, we develop a critical mind, learn to use many tools and we are used to standing before audiences to defend our point of view… My suggestion is to see yourself as the highly-educated, competent and resourceful person academia helped you become. And certainly, you can learn fast what you do not yet know.

*This interview was conducted by Associate Editor Luke R. Grinham*.

